# Optical induction of autophagy via Transcription factor EB (TFEB) reduces pathological tau in neurons

**DOI:** 10.1371/journal.pone.0230026

**Published:** 2020-03-24

**Authors:** Jessica L. Binder, Praveen Chander, Vojo Deretic, Jason P. Weick, Kiran Bhaskar

**Affiliations:** 1 Department of Molecular Genetics and Microbiology, University of New Mexico Health Sciences Center, Albuquerque, New Mexico, United States of America; 2 Department of Neurosciences, University of New Mexico Health Sciences Center, Albuquerque, New Mexico, United States of America; 3 Autophagy Inflammation and Metabolism Center of Biomedical Research Excellence (CoBRE), University of New Mexico Health Sciences Center, Albuquerque, New Mexico, United States of America; Federal University of Santa Catarina, BRAZIL

## Abstract

Pathological accumulation of microtubule associated protein tau in neurons is a major neuropathological hallmark of Alzheimer’s disease (AD) and related tauopathies. Several attempts have been made to promote clearance of pathological tau (p-Tau) from neurons. Transcription factor EB (TFEB) has shown to clear p-Tau from neurons via autophagy. However, sustained TFEB activation and autophagy can create burden on cellular bioenergetics and can be deleterious. Here, we modified previously described two-plasmid systems of Light Activated Protein (LAP) from bacterial transcription factor—EL222 and Light Responsive Element (LRE) to encode TFEB. Upon blue-light (465 nm) illumination, the conformation changes in LAP induced LRE-driven expression of TFEB, its nuclear entry, TFEB-mediated expression of autophagy-lysosomal genes and clearance of p-Tau from neuronal cells and AD patient-derived human iPSC-neurons. Turning the blue-light off reversed the expression of TFEB-target genes and attenuated p-Tau clearance. Together, these results suggest that optically regulated TFEB expression unlocks the potential of opto-therapeutics to treat AD and other dementias.

## Introduction

Among various microtubule-associated proteins (MAPs), tau (encoded by *MAPT*) predominately localizes to axons where it binds to microtubules. Tau is known to promote nucleation, stabilization, and prevent disassembly of microtubules [1]. However, tau is susceptible to many post-translational modifications [2], with phosphorylation being one of the well-studied modifications [3–5]. Upon hyperphosphorylation, tau’s affinity to microtubule decreases causing microtubules to undergo depolymerization [6], which has been the prevailing hypothesis, that such loss-of-function of tau contributes neurodegeneration [7,8]. These dissociated forms of tau can self-assemble into paired-helical filaments (PHFs) gaining further potential to aggregate as Neurofibrillary tangles (NFTs)–a classic neuropathological hallmark of Alzheimer’s disease (AD) and related tauopathies [9]. Alternatively, hyperphosphorylated and pathological tau (p-Tau) have been shown to acquire gain-of-toxic function in triggering synaptotoxicity relevant to AD [10]. Most notably, fetal tau with three-microtubule binding repeats and no N-terminal inserts (0N3R) and all isoforms of tau with T231D/S235D mutations have been previously implicated in AD. First, adult nervous system expresses all six isoforms, including 0N3R tau, at approximately same ratios as other isoforms (Reviewed in Hanger et al. [11]). Second, phosphorylation of tau on T231 and S235 has been well-established in relevance to AD [12]. Furthermore, Lu’s group has conclusively established that phosphorylation of tau on T231 causes *‘cistausosis’* [13,14], which can be restored by Pin1. Phosphorylation of T231 has also been shown to reduce microtubule-binding ability of tau [15]. Finally, a previous study has established that pseudophosphorylation of both adult (4R) and fetal (3R) tau on T231 and other related phosphorylation sites cause robust neurodegeneration relevant to AD [16]. These studies suggest that among different isoforms of tau and their phosphorylation state, 0N3R-T231D/S235D tau has direct relevance to AD pathogenesis. While AD is the most common form of tauopathy and sixth leading cause of death in the United States [17], NFT pathology is also the primary etiology in many, but rare tauopathies such as Progressive Supranuclear Palsy (PSP), Pick’s disease (PiD), Corticobasal Degeneration (CBD), Fronto-temporal Dementia and Parkinsonism linked to Chromosome-17 tau-type (FTDP-17T) and others [9]. Because of the exponential rise in tauopathy-related deaths, there is an urgent need to find intervention(s) against tauopathies.

A plausible strategy to prevent p-Tau from becoming pathological is to promote its degradation via autophagy in “*at risk*” neuronal populations. As such, there are clinical trials underway to promote clearance of tau and other aggregated proteins in patients with AD and Parkinson’s disease (NCT02947893, NCT02281474). Importantly, the NCT02281474 is also a small randomized Phase 1 study with Nilotinib (Tasigna^®^), an inhibitor of Abl non-receptor Src family kinase, on twelve participants. The expected primary outcome was on the levels of alpha-synuclein in the CSF of subjects with Parkinson’s and Diffuse Lewy Body Disease. The NCT02947893 is a phase 2 study with forty-two participants, testing the efficacy of Nilotinib on CSF levels of Abeta40/42, total Tau and p-Tau231/181, which is against the mild to moderate dementia due to AD. While the results were not very encouraging for Nilotinib on Parkinson’s disease as there were significant side effects observed and the benefit of Nilotinib disappeared upon drug discontinuation, Phase II is still underway. Besides AD, low dose of Nilotinib also being tested for Amyotrophic Lateral Sclerosis and Huntington’s disease. Moreover, impairment of autophagic processes has been implicated in several neurodegenerative disorders [18–25], which further supports autophagy’s role in clearing p-Tau and maintaining homeostasis as a potential strategy. Previous work from our group provided compelling evidence that autophagy prevents spurious inflammasome/interleukin-1β (IL-1β) activation [26,27], which when left uncontrolled, could drive tau pathology and cognitive impairment [28]. Other studies have also suggested that promotion of autophagic processing can enhance clearance of p-Tau and rescue neurotoxicity in a mouse model of tauopathy [29]. We have demonstrated that induction of autophagy via chemical (FDA approved autophagy inducing drugs, including Bromhexine) or genetic Transcription Factor EB (TFEB) means lead to the clearance of inflammation-induced p-Tau in neuronal cells [30]. Notably, Phase 2 clinical trial is underway for the drug called Ambroxol (a mucolytic active product of the prodrug Bromhexine) against the CSF levels of tau and α-synuclein after observing encouraging CSF availability of the drug and notable improvement in the unified Parkinson’s disease rating scale among participants (unpublished, presented at the 2018 AD/PD conference). The TFEB regulates transcription of an entire CLEAR (*Coordinated Lysosomal Expression and Regulation*) network, which consists of a consensus site predominately found in the promoter regions of autophagy-lysosomal genes [31,32]. Thus, when TFEB localization is nuclear, it leads to a robust increase in lysosome biogenesis, and results in accelerated degradation of autophagic substrates [30,33]. Phosphorylation of Ser211 in TFEB by mammalian target of rapamycin complex 1 or mechanistic target of rapamycin complex 1 (mTORC1) is one of the key regulators of nuclear localization, as the S211 phosphorylation prevents TFEB from entering into the nucleus [34]. However, the limitation of pro-autophagy studies is their focus on the continual activation of autophagy. While autophagy is generally thought to promote survival as discussed above, under certain conditions sustained autophagic-flux can lead to cell death [35].

Furthermore, prolonged activation of autophagy proteins (e.g., LC3 and BECN1) and vacuoles in response to ischemic stroke/reperfusion *in vivo*, or oxygen-glucose deprivation (OGD) *in vitro* lead to significant cell death [36]. Interestingly many autophagic processes do not significantly affect cell health until days after the injury, indicating that prolonged activation is critical for cell death to occur [37,38]. Another example, constitutive activation of the δ2 glutamate receptor was demonstrated to cause Purkinje cell death in Lurcher mice via activation of autophagy [39]. Thus, for elderly tauopathy patients with co-morbid conditions such as ischemia and vascular dementia, sustained activation of autophagy could exacerbate cell death. Therefore, it is crucial to develop tunable systems to turn-on/turn-off autophagy in neurons with optimum spatio-temporal control.

Historically, chemically regulatable gene expression systems, such as the tetracycline-regulated transcription system [40], has been the most widely used approach to manipulate the expression of gene of interest. Recently, the use of plant flavoproteins (light stimulation) have been engineered to control mammalian transcription factor activity [41]. To date, optogenetic technology has been primarily utilized to alter membrane excitability in neurons using microbial opsins that gate ion channels [42]. However, an underutilized application of this technology is that of reversible optical regulation of transgene expression [41]. A previous study has utilized a regulatable version of EL222, a bacterial Light-Oxygen-Voltage (LOV) protein that has been shown to bind to DNA when activated with the blue-light [43–47]. This system has been shown to induce transcription of target genes with >100 fold dynamic range and rapid activation (<10 s) and deactivation (<50 s) kinetics [43]. While this system has been tested in various mammalian cells and zebrafish embryos, its functional utility in a human disease model system remains untested.

In the case of autophagy, which requires the coordinated expression and function of a host of proteins, optical induction of a key master transcription factor (such as TFEB) serves as a best target for the functional validation of optogenetics system in human disease models. Here we optimized an optical induction system based on EL222- light-responsive bacterial transcription factor [43] to drive TFEB expression in different cell-based models of tauopathy. For the first time, our group has shown that optically controlled TFEB efficiently expresses in human AD neurons, up-regulates TFEB target genes, and efficiently reduces multiple pathological forms of tau.

## Materials and methods

### Vector construction

All constructs (Table 1) were cloned using NEB HIFI Assembly Kit (NEB # E5520S) with restriction enzymes and PCR amplification. Briefly, the original episomal plasmids gifted by Motta-Mena et al [43], (pVP-EL222 and pGL4-C120-mCherry) were cloned into different backbones with subsequent promoters and /or gene of interest; pN1-CMV-TFEB-GFP (Addgene # 38119). Newly cloned episomal plasmids were then additional cloned into lentivector backbone, pGF1-*Nfkb*-EF1-Puro (Systemsbio # TR012PA-P). Q5^®^ Site-Directed Mutagenesis Kit was used to make site-directed point mutations (S142A and S211A) in TFEB gene (NEB # E0445S). All Tau constructs used; 1) pRC/CMV - 0N3R-tau (human tau with three microtubule-binding repeats with no N-terminal inserts); 2) 0N4R-tau (human tau with four microtubule-binding repeats with no N-terminal inserts); 3) 0N4R-P301L (human tau with four microtubule-binding repeats with P301L FTDP-17T mutation); 4) 0N3R-T231D/S235D. See Table 1 for all cloned vectors and their corresponding names.

**Table 1:** Light responsive plasmids.

Name	Description	References/ Source
pGL4-SV40-VP-EL222	Bacteria Transcription Factor, EL222, LOV domain.	**Motta-Mena et al**
pC120-MCH	mCherry reporter	**Motta-Mena et al**
pC120-FLuc	Firefly Luciferase reporter	**Motta-Mena et al**
pN1-CMV-TFEB-GFP	Constitutive TFEB- GFP reporter	**Addgene #38119**
pN1-CMV-TFEB(S211A)-GFP	Constitutive TFEB with (S211A) mutation- GFP reporter	
pN1-LRE-TFEB3xFLAG WT	LRE-Flag reporter	**Light response element (generated for the present study)**
pN1-LRE-TFEB(S142A)3xFLAG	LRE-Flag reporter	
pN1-LRE-TFEB-GFP WT	LRE-GFP reporter	
pN1-LRE-TFEB(S211A)-GFP	LRE-GFP reporter	
pGF1-LRE-TFEB(S211A)-GFP	Lenti-LRE-TFEB-GFP reporter	
pN1-CMV-EL222	LAP, CMV promoter, Sv40 NLS N term	**Light-activated protein**
pN1-CMV-EL222-_2x_NLS	LAP, CMV promoter, Sv40-NLS, and cMyc NLS	
pGF1-CMV-EL222-_2x_NLS	Lenti-LAP	

### Cell lines

HEK293T and Neuro-2a (ATCC # CRL-3216 and #CCL-131, respectively) cells were maintained at 37°C in 5% CO_2_ in DMEM supplemented with 10% FBS, 5% penicillin/streptomycin, and grown in 24-well plates. For transient transfections, cells were split the day before ~ 1–4 × 10^5^ cells/well, therefore 70–80% confluence the following day. Before transfection, media was replaced with phenol red free media, (FluoroBrite DMEM; ThermoFisher # A1896701). Cells were then transfected with Lipofectamine 2000 (Invitrogen) as per company’s protocol. Dilutions of various plasmid concentrations were as followed for a 24-well plate; pLAP’s–(2000ng/μL), pLRE’s–(500ng/μL), pCMV-TFEB’s–(500ng/μL), pCMV-hTau’s–(1000ng/μL), pCLEAR-FLuc–(500ng/μL) and maintained the LRE to LAP ratio at 1:4.

### Induced pluripotent stem cells

sAD2.1 [48]—Coriell # GM24666, (iPSCs from Fibroblast NIGMS Human Genetic Cell Repository Description: *ALZHEIMER DISEASE; AD* Affected: *Yes*. Gender: *Male*. Age: *83 YR (at sampling)*. Race: *Caucasian*.)

Briefly, iPSCs were maintained in mTESR plus the supplement (StemCell # 85850) Neuron differentiation followed the StemCells neuronal differentiation kit/protocol; (StemCell #05835, #05833, #08500, #08510). Later medium was changed to BrainPhys^™^ without Phenol Red (StemCell #05791) for optical induction. (Neural progenitor cells seeded at 1.5x 10^4^ cells/cm^2^ for maturation).

### Light induction

Twelve hours post-transfection, an in-house blue LED device (465 nm, strip of LEDs glued to PCB board; Amazon) was placed 8 cm or 16 cm above the plate. Note, the constraints of the light source also had to be altered (twice the distance than our cell lines; 16 cm) due to higher sensitivity of iPSNs to the blue-light and the heat it produces, compared to N2a cell lines. The intensity of the light received by cells was measured to be to 8 W/m^2^; as previously reported by [46]. Verified, using the LI-190 Quantum Sensor and LI-250A light meter (LI-COR Biosciences). The LED strips were connected to SLBSTORES 3528 5050 12V DC Mini Remote Controller (Amazon) for variations of on/off patterns to best match a cycle of 20 s ‘on’ and 60 s ‘off’ as recommended per Motta-Mena et al [43]. The control plate was kept in a PCB blackout box with breathable air slots, (a shelf in the incubator, above and away from the light source shelf). For transiently transfected cells, 24 h post-transfection, samples were collected/fixed for analysis.

### Lentivirus production and luciferase assay

Using HEK293T’s, seeded in 100 mm plates. Lentiviral Transgenes were cloned into the pGF1-EF1-Puro backbone. Lentiviral packaging vectors: pMD.2, pPAX2 (Invitrogen cat. no. K4975-00). Cells were transfected with plasmid mix using CaPO_4_ precipitation method. After 48 h interval, the viral supernatant was then filtered through 0.45 μm membranes and mixed overnight with cat# 631232 Lenti-X^™^ Concentrator. The next day, samples were centrifuged at 1,500 x g for 45 minutes at 4°C. An off-white pellet is then resuspended in subsequent media, (for iPSNs, the pellets were resuspended in neurobasal media). Lentiviral titer was measured using cat# 631280 Lenti-X^™^ GoStix^™^ Plus. Lentiviral Transduction on iPSNs—an IFU of 1x10^6^/mL were added to the neurons to make ~MOI = 2. We transduced sAD2.1 neural progenitor cells 24 h after plating on poly-ornithine/laminin coated coverslips following StemCell^®^’s maturation protocol. Subsequently, two weeks after transduction, (Day 40) iPSNs are subjected to light stimulation (12 h) or kept in the dark, samples were then collected/fixed for analysis.

For Firefly luciferase activities, 4XCLEAR-luciferase reporter plasmid #66800, purchased from Addgene. D-Luciferin, Potassium Salt (ThermoFisher # L2916) was reconstituted in water and was added (1:100) to each well, 3–4 min after addition of substrate, 24-well plate samples were analyzed through the IVIS Lumina Series II with system software. (n = 1 refers to an entire 24 well plate, and 6 wells individually calculated per control).

### Western blotting analysis

Cells were lysed by RIPA buffer (Thermo #89900), incubated on ice for 30 min then centrifuged at 20,000 × g for 15 min. Cell lysate supernatants were then sonicated for 20 sec at 30%, then subjected to SDS-PAGE usage, transferred to PVDF membranes and detected using the ECL method (Pierce). Protein levels were quantified using ImageJ (National Institute of Health). Antibodies included; Tau12, Actin, GAPDH, GFP, AT8, AT180, and VP16. Source and dilutions of the antibodies are used are provided in Table 2. Unless otherwise noted, corresponding secondary antibodies conjugated to horseradish peroxidase were utilized at 1:10,000 dilutions.

**Table 2:** Antibodies and the sources used for western blot and immunocytochemical analysis.

Antibody	Species	Company and Catalog #	Dilutions
**FLAG**	Rabbit/mouse	Abcam ab1162, ab49763	1:5000 (WB) 1:500 (IF)
**GFP**	Chicken/mouse	Abcam ab13970, ab1218	1:1000 (WB) 1:1000 (IF)
**VP16**	Rabbit	Abcam ab4808	1:1000 (WB) 1:250(IF)
**Beta-Tubulin**	Rabbit/chicken	Abcam ab18207, Abcam ab18207	1:10,000 (WB)
**AT180**	Mouse	Thermo Scientific, MN1040	1:5000 (WB) 1:500(IF)
**AT8**	Mouse	Thermo Scientific, MN1020	1:5000 (WB) 1:500(IF)
**GAPDH**	Mouse	Millipore, CB1001-500UG	1:20,000 (WB)
**Tau12**	Mouse	Abcam, ab74137 Millipore, MAB2241	1:20,000 (WB)

### Immunocytochemical analysis

Cells were plated on coverslips coated with laminin, once cells were ready for fixation, they were fixed in 4% PFA, blocked with 0.2% triton and 10% donkey serum (DS), incubated in primary overnight in 4°C (5% DS), secondaries were incubated for 1h at RT. Incubated in DAPI for 10 min, and mounted to slides using Fluoromount-G^™^ (Cat# 00-4958-02; Thermo Fisher Scientific). Immunofluorescence confocal microscopy was carried out using Zeiss LSM 510 Meta microscope. Quantitative morphometry and profile analysis were performed using ZEISS ZEN imaging Software. Antibodies included; Tau12, VP16, GFP, AT8, AT180, and beta-tubulin. Source and dilutions of the antibodies are used are provided in Table 2. Unless otherwise noted, corresponding secondary antibodies conjugated to horseradish peroxidase were utilized at 1:10,000 dilutions. For the quantitative morphometry in N2a cells, number of DAPI, VP16 and GFP positive cells per 40x field were counted and the percentage ratio of GFP/VP16 positive cells were scored. Five random field per technical replicate were quantified. For the analysis, percentage of GFP/VP16 positive cells in at least three technical replicates and three biological replicates (see below under statistics) were quantified. In case of sAD2.1 iPSN immunocytochemistry, average intensity of TFEB (S211A)-GFP, AT8 and AT180 immunoreactive areas were quantified in five random fields per condition and repeated in at least three biological replicates. Average intensity from all five fields per technical replicate were averaged and plotted as a data point.

### Gene expression analysis

RNA from cells was extracted using the TriZOL reagent as described by the manufacturer (Thermo Fisher Scientific). Total RNA (20 ng/μL) was converted to cDNA using the High Capacity cDNA Reverse Transcription kit (Thermo Fisher Scientific) and amplified using specific TaqMan assays (catalog # 4331182; Thermo Fisher Scientific). GAPDH (catalog # 4352339E, Thermo Fisher Scientific) was used as a housekeeping gene for normalization. qRT-PCR assays were run on the StepOnePlus^®^ Real-Time PCR System (Thermo Fisher Scientific) and the statistical analyses were performed using Prism.

### Cellomics^®^-based high-content imaging analysis

Cells were plated in 96 well plates transiently transfected with pCMV-T231D/S235D (phosphorylation-mimicking tau), pCMV-LAP2xNLS, and pLRE-TFEB(S211A)-GFP. Twenty-four hours later, cells were incubated with conditioned medium from BV2’s, as previously described, then subsequently induced with light (470 nm) for 12 hours. Cells were fixed in 4% PFA, blocked with 0.2%triton and 10% donkey serum, incubated in primary antibody for one hour at RT (5% DS), followed by secondary antibodies for 1hr at RT. Incubated in DAPI for 10 mins and analyzed through Cellomics^®^ high content microscopy. For the automated quantification via Cellomics high-content microscopy, the Cellomics software was programmed to set the criteria for cell boundary, nucleus and the remaining field as cytosol. A threshold for automated scoring was set at 200 cells/per condition (or per well). GFP positive cells (normalized to CMV-TFEB (S211A)-GFP) and Tau12 mean intensity was scored and plotted.

### Statistics

Unless otherwise indicated, all cell culture experiments were performed in at least three technical replicates and n = 3 (minimum) to n = 9 (maximum) biological replicates, which included performing experiments in cells grown in different plates on a different day. Results from three technical replicates and one biological replicate was considered as n = 1. Comparisons between the two groups were done via unpaired *t* test; comparisons between multiple treatment groups were done via one-way or two-way analysis of variance (ANOVA) with indicated multiple comparisons post-hoc tests. All statistical analyses were performed using GraphPad Prism^®^.

## Results

### Cytomegalovirus (CMV) promoter and the nuclear localization signal (NLS) sequence derived from cMyc shows robust gene expression with light

**Fig 1:**
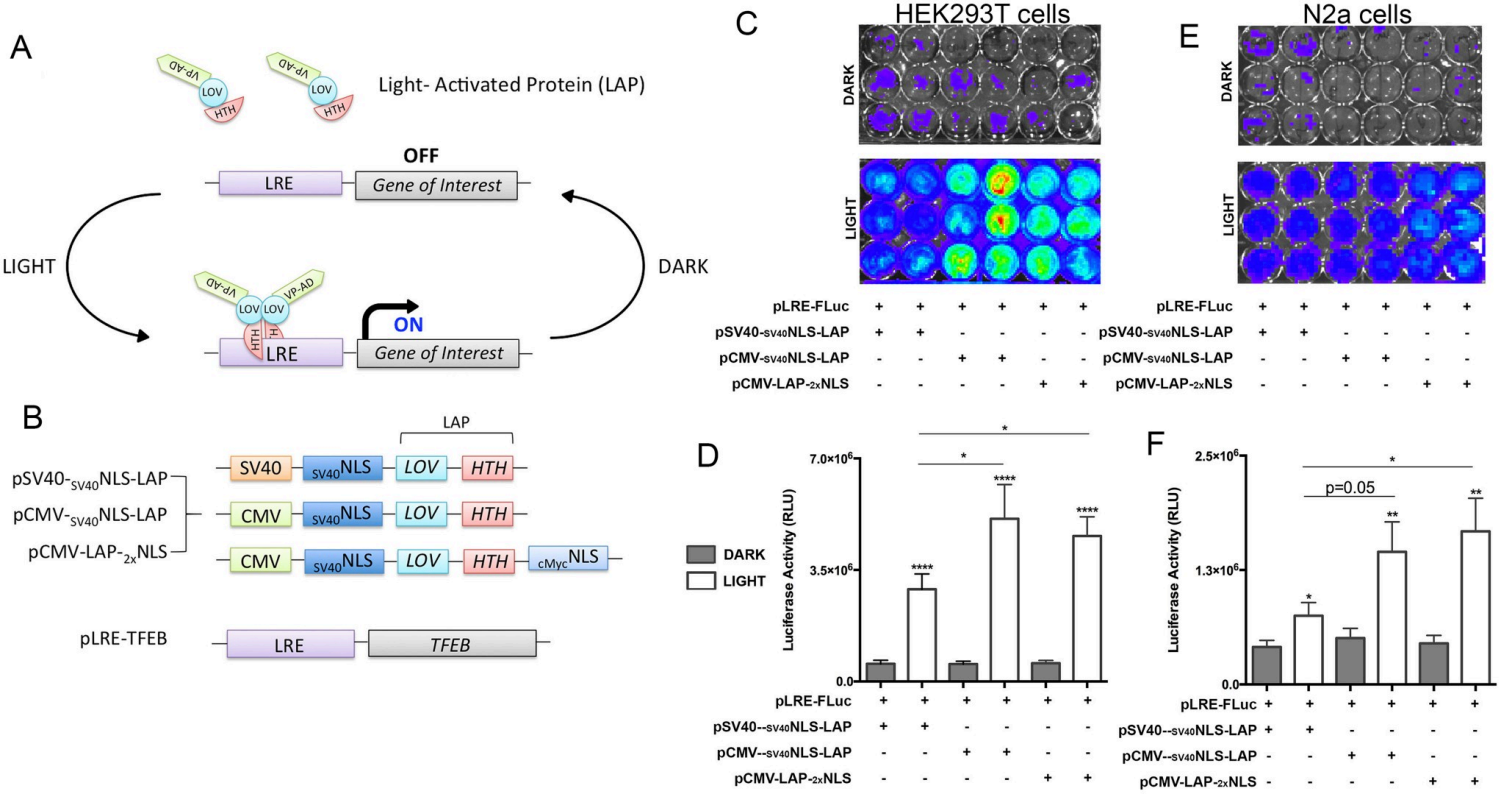
Optogenetic gene expression system in neuronal cell line. A. Schematic of previously established gene expression system derived from an EL222 bacterial transcription factor, termed Light-Activated Protein (LAP). B. Schematic of optimizations made to the LAP construct for successful neuronal transfection/induction as well as TFEB cloned into the LRE construct. C-F. Quantitative comparison of various versions of LAP constructs using pLRE-Firefire Luciferase reporter, (pLRE-FLuc) in HEK293T cells (C and D) and N2a neuroblastoma cells (E and F), measuring luciferase activity units (RLU) via radiance levels detected by IVIS (mean ± s.e.m, unpaired Student’s *t* test or one-way ANOVA with Tukey multiple comparison test, ****p<0.0005 n = 5).

Intending to optically control autophagy at the transcriptional level, we chose a light-inducible gene expression system that utilizes an engineered bacterial transcription factor EL222, containing a Light-Oxygen-Voltage (LOV) [43–47] protein and N-terminal VP16 transcriptional activation domain. The corresponding DNA binding region to EL222 was previously optimized with five copies of a specific EL222 DNA-binding region, [Clone 1–20 base pairs (C120)_5_] [43] (Fig 1A). This consensus site acts as a promoter region for the EL222 binding and drives the expression of any genes inserted downstream of C120 allowing for transient expression of the transgene due to relatively fast reductions in expression upon cessation of light exposure (Fig 1A).

First, we verified that co-transfection of HEK293T cells with both the pVP-EL222 (referred to as the ‘light activated protein’ or ‘LAP’) and the pC120-Fluc (referred to as the ‘light-response element’ or ‘LRE’), resulted in optically-induced expression of the firefly luciferase reporter. We observed robust luciferase expression and activity driven by the LAP-LRE interaction in HEK293T cells (Fig 1C and 1D). However, luciferase expression and activity were more than a two-fold lower in Neuro 2a (N2a) cells compared to HEK293T cells (Fig 1C, 1D, 1E and 1F). In an attempt to optimize the LAP-LRE system for gene expression in neurons we replaced the SV40 promoter with a stronger CMV promoter [49,50] (Fig 1B). We also included an additional cMyc nuclear localization signal (_1x_NLS or _2x_NLS) sequences [51,52] (Fig 1B). The addition of the different promoter was sufficient to significantly improve luciferase expression upon blue-light illumination compared to Dark controls in both HEK293T and N2a cells (Fig 1C–1F). We also observed a different degree of luciferase expression with the different promoter and dual NLS combinations, pCMV-LAP-_2x_NLS, showing the most robust induction of luciferase expression in N2a cells (Fig 1C–1F). Due to the notable light-induced transgene expression by pCMV-LAP-_2x_NLS in both cell lines, we used this LAP construct for all optical experiments.

### TFEB clears multiple forms of pathological tau with equal efficiency in cellular models of tauopathy

**Fig 2:**
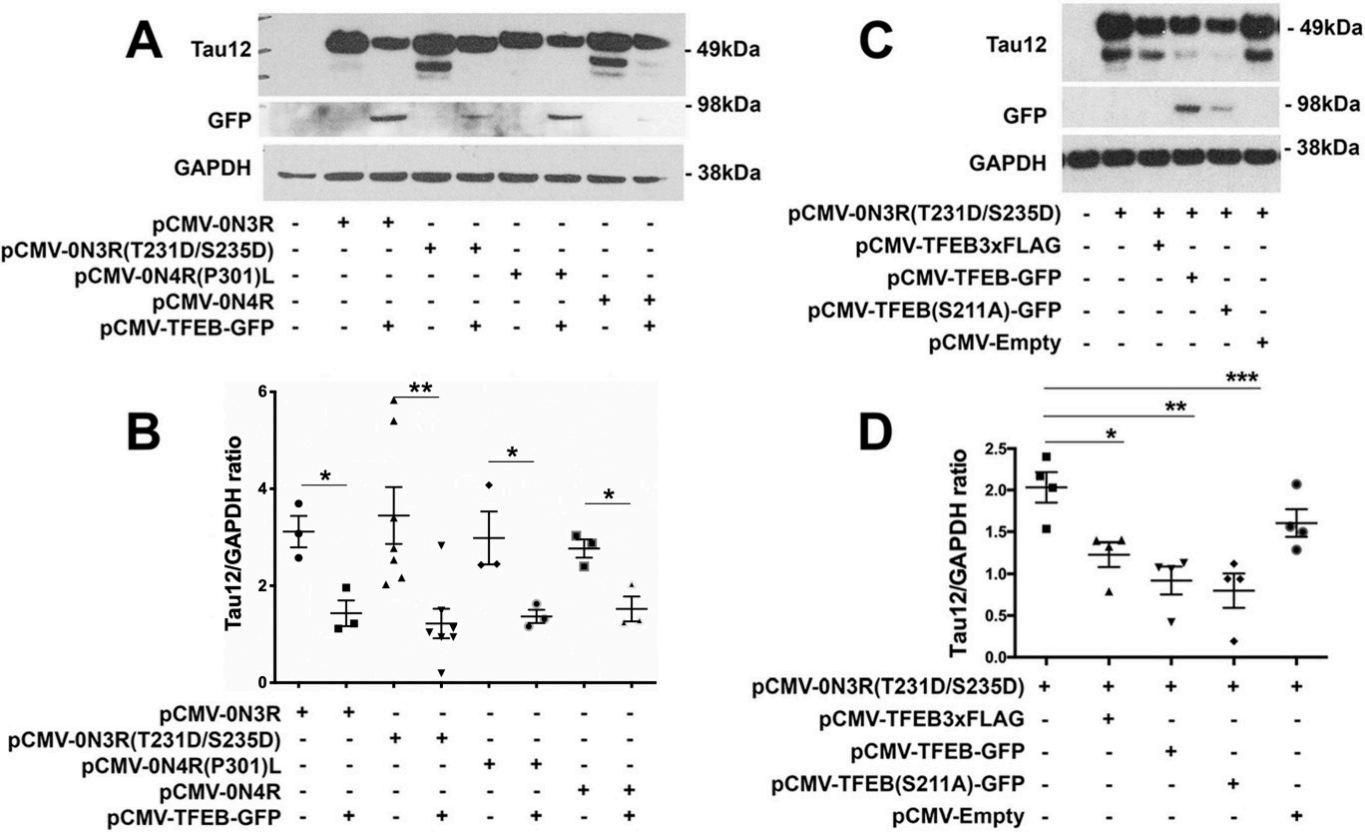
TFEB differentially targets various forms of pTau. A-B. Western blot and quantification showing significant reduction in various forms of tau via WT– 0N3R, (0N3R) T231D/S235D, (0N4R) P301L, and WT– 0N4R with the addition of constitutive overexpression of TFEB activity. Results indicated most forms of tau are equivalently reduced by TFEB, however (0N3R) T231D/S235D shows highest significance in expression and reduction. Total tau/GAPDH ratio (mean ± s.e.m, unpaired Student’s *t* test, ** p<0.01, n = 3). Note only the ~49 kDa, but not the low molecular weight, Tau12^+^ band was quantified for the analysis. C-D. Western blot and quantification showing significantly reduced (0N3R) T231D/S235D with the addition of various forms of constitutive TFEB overexpression; pCMV-TFEB-3xFLAG, pCMV-TFEB-GFP, pCMV-TFEB (S211A)GFP. Results indicate pCMV-TFEB (S211A) GFP holds the best yield in total tau reduction. Total tau/GAPDH ratio (mean ± s.e.m, one-way ANOVA with Dunnett’s multiple comparison test, *p<0.05; **p<0.01; ***p<0.005 n = 4).

To test the ability of optically induced transgene expression to clear p-Tau, we chose TFEB, which is a well-established regulator of autophagy, and previously implicated in clearing tau via constitutive activation [29,30,53,54]. As a first step, we decided to confirm whether TFEB can clear p-Tau and determine whether TFEB can target multiple forms of p-Tau via autophagic flux in neuronal cells. The *MAPT* gene in humans encodes six different isoforms that differ based on inclusion or exclusion of exons 2, 3 and 10 [55]. Exon 10 encodes the second microtubule binding repeat, thereby resulting in tau with either three (3R) or four (4R) microtubule binding repeats of 31–32 amino acids in the C-terminal half of tau [55]. Exons 2 and 3 encode one (1N), two (2N), or zero (0N) amino terminal inserts of 29 amino acids each in the N-terminal half of the protein [55]. In normal adult brain, the relative amounts of 3R tau and 4R tau are approximately equal. However, in many neurodegenerative tauopathies, the 3R:4R ratio is often altered [9]. Besides altered isoform ratios, post-translational modifications such as phosphorylation can also affect tau’s function and contribute to disease pathogenesis. We tested if TFEB can clear following types of p-Tau: **(1)** 0N3R –non-mutant tau, when over-expressed can lead to Pick’s Disease (PiD) [56], **(2)** 0N3R (T231D/S235D) tau, which mimics hyperphosphorylation on T231/S335 sites and is known to disrupt tau’s interaction with microtubules [57], **(3)** 0N4R –non-mutant tau, but over-expression can lead to progressive supranuclear palsy (PSP) [58], and **(4)** 0N4R-P301L mutant tau, which cause FTDP-17T [59,60]. Others and our group have previously shown that TFEB-induced autophagic flux degrades p-Tau via beclin-1 dependent autophagy pathway [30]. However, it is unclear whether TFEB can target and clear various pathological forms of tau. Here we co-transfected N2a cells with a 1:1 [DNA] ratio; constitutive TFEB expressing vectors with each individual tau constructs mentioned above, 0N3R, 0N3R(T231D/S235D), 0N4R, or 0N4R-P301L. As revealed by western blot, TFEB expression caused a significant reduction in all forms of tau in N2a cells (Fig 2A and 2B), with T231D/S235D phosphorylation-mimicking tau showing the most significant reduction (Fig 2A and 2B). Together, these results suggest that TFEB can consistently clear different types of p-Tau in neuronal cells. Furthermore, since the T231 mutation causes a potent neurotoxic conformation called *cis*-p-Tau (or ‘*Cistauosis*’, as a result of phosphorylation of tau at T231) [22,61,62], TFEB’s role in significantly reducing T231D/S235D levels supports the therapeutic potential of targeting TFEB against tauopathies. Next, we also determined whether or not TFEB with different tags (FLAG versus GFP) would affect its ability to clear p-Tau via autophagy. Co-expression of T231D/S235D tau with either pCMV-TFEB3xFLAG or pCMV-TFEB-GFP showed that GFP tagged TFEB has better efficiency in inducing p-Tau reduction than 3xFLAG tagged TFEB (Fig 2C and 2D). While this observation is still unclear, we speculate that due to GFP’s tendency to dimerize [17,19] as well as TFEB’s homodimerization or heterodimerization [18] characteristics, combined/fused together potentially enhances TFEB’s ability as a transcription factor as well as stability efforts.

We observed that TFEB-GFP distribution appeared homogenous throughout cells, indicating that overall nuclear entry of the TFEB was relatively low. To achieve better nuclear entry of TFEB, we tested S211A mutation in TFEB, which was previously shown to prevent phosphorylation by mTORC1 [34] thereby facilitates TFEB’s nuclear entry. Given that the transcriptional promotion of genes in the CLEAR network requires nuclear localization of TFEB, we next assessed the effects of S211 phosphorylation in TFEB in clearing mutant p-Tau. We observed robust reduction of T231D/S235D mutant p-Tau when they were co-expressed with TFEB (S211A) (Fig 2C and 2D). Together, these results suggest that genetically facilitating the nuclear entry of TFEB does provide an added advantage in enhancing the autophagic clearance of T231D/S235D tau.

### Optogenetically expressed TFEB activates CLEAR network genes in neuronal cells

**Fig 3:**
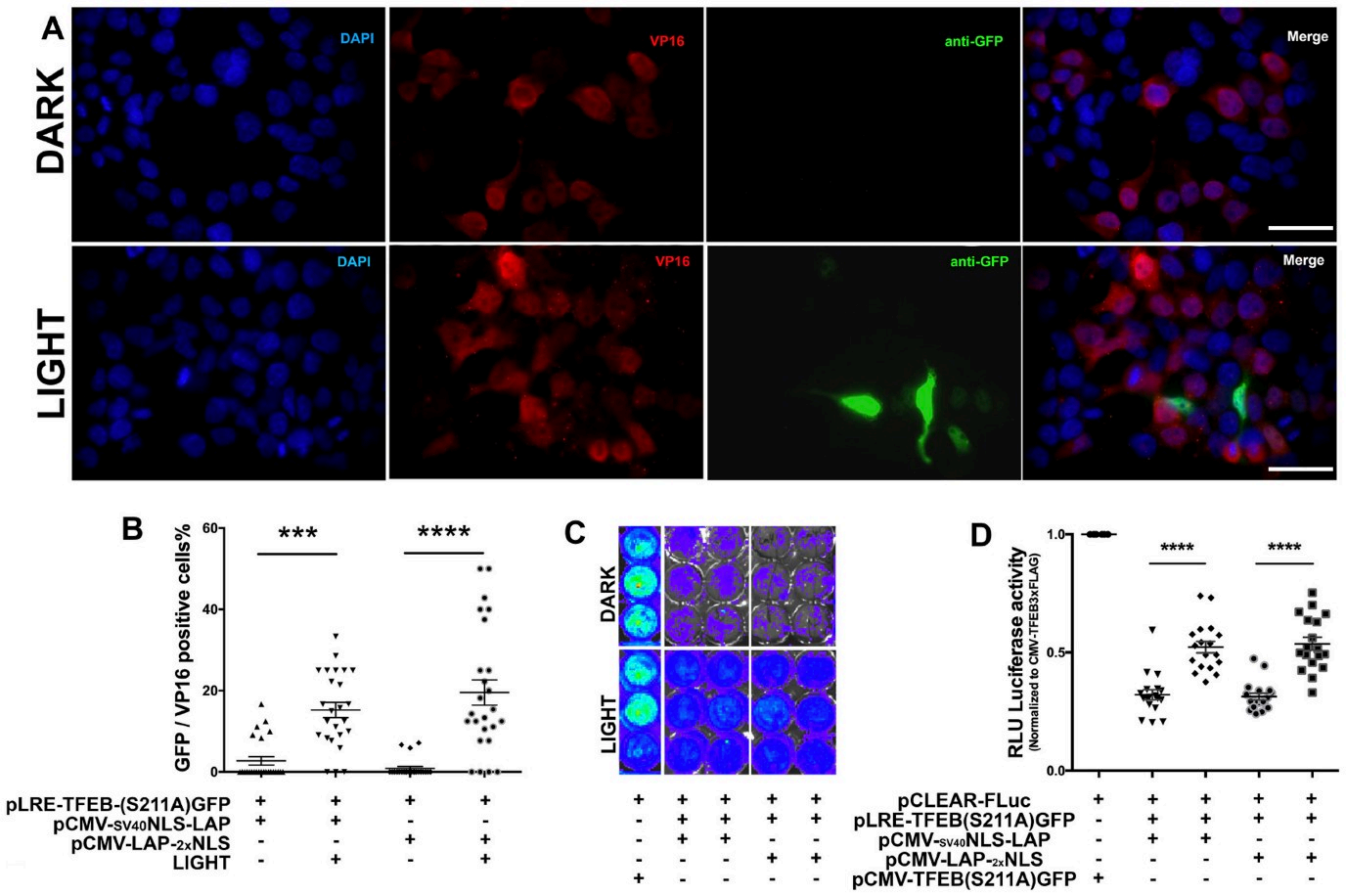
Optogenetic TFEB induction in neuronal cell line and CLEAR activity readout. A-B. Quantitative immunocytochemistry showing significant increase in TFEB expression in Light control vs Dark, comparison of various versions of LAP constructs using pLRE-TFEB-(S211A) GFP. Scale bar: 20 μm C-D. Quantitative comparison of various versions of LAP constructs using pCLEAR-Firefly Luciferase reporter, (pCLEAR-Fluc) in N2a cells measuring luciferase activity units (RLU) via radiance levels detected by IVIS (mean ± s.e.m, unpaired Student’s *t* test, ****p<0.0005, n = 4).

To determine the efficiency of optogenetically-driven TFEB (Opto-TFEB) in N2a cells, we co-transfected N2a cells with either pCMV_SV40_NLS-LAP or pCMV-LAP-_2x_NLS and pLRE-TFEB (S211A)-GFP plasmids. The cells were stimulated with blue light for 12h and immunostained to detect the levels of LAP (VP16) and LRE (TFEB (S211A)-GFP). In the initial characterization studies, we tested the Opto-TFEB at three different time points (6h, 12h and 18h) in N2a cells. Our results suggested that 12 h time point showed optimum levels of light-induced expression of TFEB-FLAG (not shown). Substitution of the SV40 promoter for a CMV promoter, along with the addition of a second cMyc NLS resulted in a significant increase of TFEB expression (revealed by GFP signal) with light stimulation compared to the ‘Dark’ control (Fig 3A and 3B). As expected, the VP16 staining was detectable and localized primarily to the nucleus in cells expressing pCMV-LAP-_2x_NLS (Fig 3A).

As mentioned, many of the target genes activated by TFEB have been identified, and all carry the consensus CLEAR motif (^5’^GTCACGTGAC^3’^) in their promoter regions [31]. To determine whether Opto-TFEB is functionally active, we used a firefly luciferase (Fluc)-based reporter assay to assess the expression of CLEAR-dependent gene [63]. The pCLEAR-FLuc plasmid consists of four replicates of the CLEAR consensus sequence upstream of the luciferase gene, thus representing TFEB transcriptional activity. We transiently co-transfected pCLEAR-FLuc with pLAPs, and pLRE-TFEB(S211A)-GFP in N2a cells and stimulated with blue light overnight (12 h). Then the cells were treated with D-luciferin, and culture plates were immediately imaged using luminometer to detect light output from the oxidation of D-luciferin as a measure of luciferase activity. As expected, the CMV-driven constitutively active TFEB produced the highest levels of CLEAR-luciferase signal (Fig 3C and 3D) that was present even in cells maintained in the Dark control condition. Interestingly, we observed significantly higher levels of CLEAR-luciferase signal in cells that expressed Opto-TFEB and were light exposed, but minimal CLEAR-luciferase signal from samples maintained in the Dark (Fig 3C and 3D). Together, our results suggest that Opto-TFEB expression is induced by blue light exposure and can functionally activate transcription of downstream targets in the CLEAR network.

### Opto-TFEB reduces pathological tau in neuronal cells

**Fig 4:**
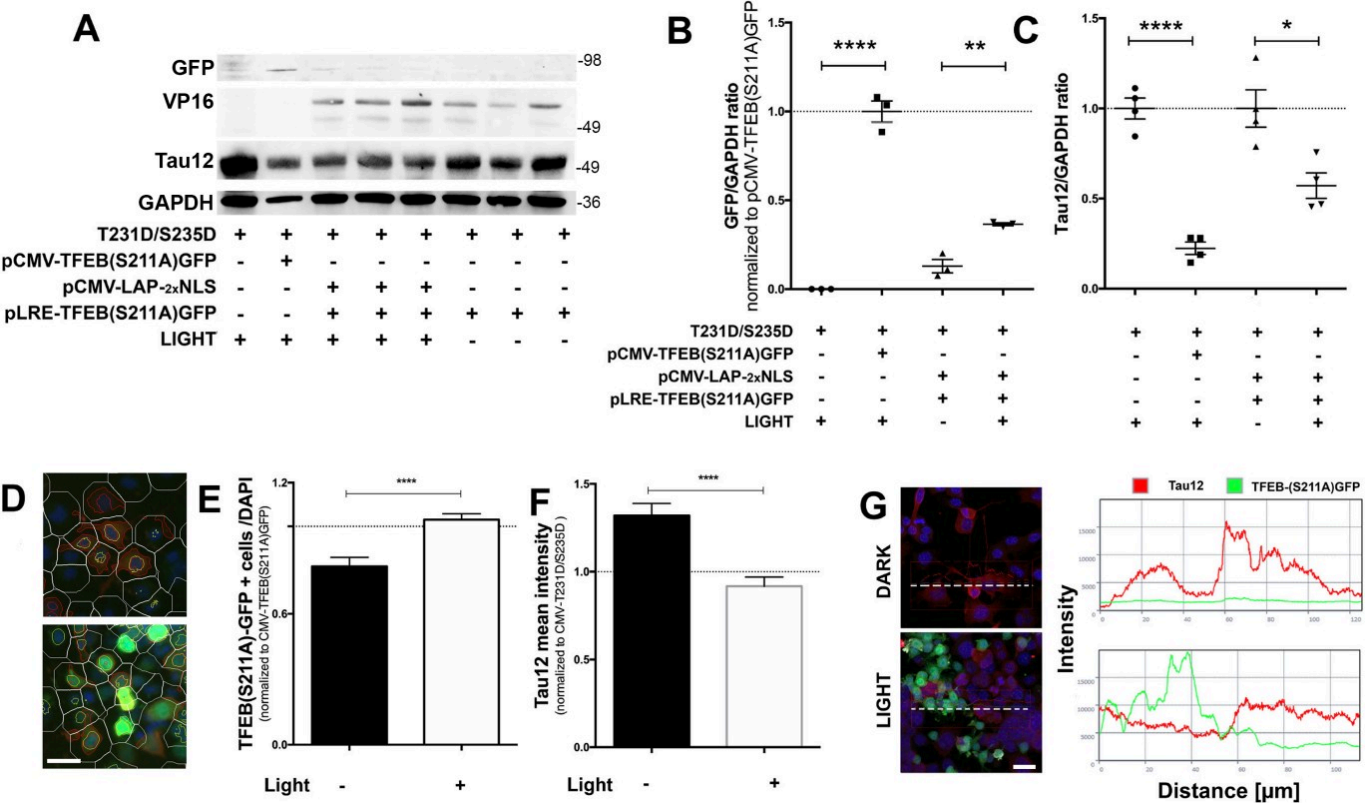
Optogenetic TFEB induction in N2a neuronal cell line reduces neuronal pathological mimicking tau. A-C. Western Blot analysis showing overall reduction in the tau levels (Tau12) when Opto-TFEB is expressed via light stimulation compared to dark. D-F. Cellomics^®^-based high-content imaging analysis of the effects of Opto-TFEB on total tau levels in Dark and Light conditions. Cells were automatically identified based on nuclear staining (DAPI), then cells were selected for positive nuclear green fluorescence (TFEB(S211A)GFP) to further analyze for Tau12 (RED) intensity levels within 100 pixel radius per cell. Briefly, white lines represent cell boundaries, red lines represent positive cytosolic Tau12, and yellow lines indicate nuclear TFEB (S211A)GFP-positive cells, then subjected by automated image analysis. G. Representation of colocalization profile for Tau12 (red) and LRE-TFEB (S211A) GFP (green) analysis. Quantitative confocal immunocytochemistry using N2a cells overexpressing human 0N3R-T231D/S235D tau show lack of colocalization of optogenetically induced TFEB expression with Tau12 positive cells. Quantitative morphometric data (mean + s.e.m, unpaired Student’s *t* test, ****p<0.0001, n = 3). Scale bars: 10 μm (in D) and 20 μm (in G).

If light-induced Opto-TFEB can bind the CLEAR motif and drive transcriptional regulation, we hypothesized that it would be sufficient to induce autophagic flux and reduce levels of misfolded p-Tau. We first overexpressed human tau carrying the 0N3R-T231D/S2345D double mutation along with pCMV-LAP_2x_NLS and pLRE-TFEB (S211A)-GFP in N2a cells. Analysis of TFEB(S211A)-GFP and Tau12 through western blot revealed statistically significant increase in TFEB expression (Fig 4A and 4B) and reduction in the levels of total tau (Tau12) (Fig 4A–4C) in light-exposed cells. Confirmatory, unbiased quantitative morphometry analysis for Tau12 levels using high-content, automated Cellomics^®^ high content microscopy, revealed a significant decrease in the overall Tau12 intensity in light-exposed Opto-TFEB^+^ cells compared to Dark controls (Fig 4D and 4F). Confocal analysis further confirmed that the fluorescence signals for Tau12 and GFP (from TFEB (S211A)-GFP+ cells) were mutually exclusive and non-overlapping (Fig 4G). Together, these results demonstrate that light-induced expression of TFEB is capable of reducing overexpressed phospho-mimicking (T231D/S235D) tau levels in neurons.

**Fig 5:**
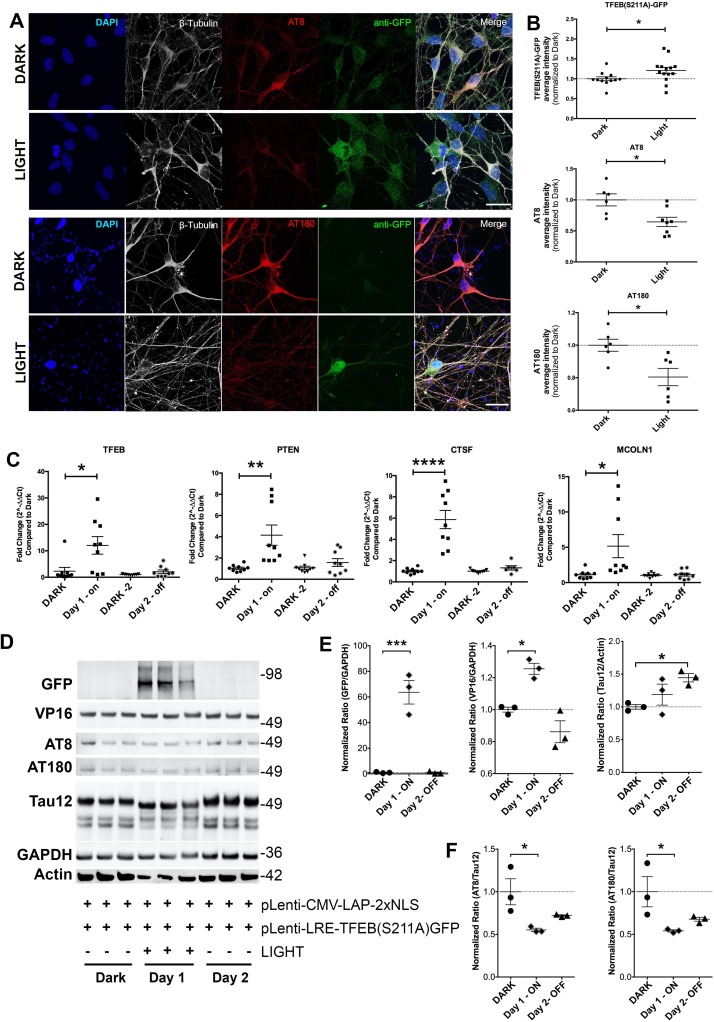
Optogenetic TFEB clears pTau in human induced pluripotent stem cells derived into neurons (iPSNs). A-B. Quantitative immunocytochemistry showing significant increase in TFEB expression with subsequent lower levels of p-Tau (AT8 and AT180) within *beta*III-tubulin (neurons) in Light control compared to Dark, using viral-particle versions, pGF1-CMV-LAP-2xNLS and pGF1-LRE-TFEB-(S211A)GFP (Scale bars: 20 μm. Mean ± s.e.m, unpaired Student’s *t* test, *p<0.05, n = 8). C. Two-day timeline using qRT-PCR analysis of TFEB gene expression and TFEB targets (PTEN, CTSF, and MCOLN1). Compared to Dark, each sample was taken 24 hours of subsequent time-point. On Day-1, 12-hour light stimulation; Day-2 from same sample, light was off. Data shown are mean ± s.e.m, unpaired Student’s *t* test, *p<0.05; **p<0.01; ****p<0.001, n = 8). D-F. Corresponding to qRT-PCR time-point samples, western blot and quantification showing significantly increased in GFP (TFEB) levels and congruently reduced p-Tau (AT8 and AT180) with the transduction of viral optogenetic TFEB and subsequent light stimulation. Note that Tau12/Actin, but not Tau12/GAPDH, ratio was significantly altered on Day 2 compared to Dark control levels. (mean ± s.e.m, one-way ANOVA with Dunnett’s multiple comparison test, *p<0.05; ***p<0.0005, n = 3–6).

To test the efficacy of this system in human-relevant model system, we tested Opto-TFEB in induced pluripotent stem cells (iPSC) line from a patient with sporadic AD (sAD2.1) [48]. As previously described [48], the iPSC-derived neurons (iPSNs- sAD2.1 line) displayed robust hyperphosphorylation on Ser202 and Thr231 sites (positive for AT8 and AT180; Fig 5A and 5D). To assess the efficacy of Opto-TFEB in sAD2.1 cells we created lentiviral Opto-TFEB constructs (pGF1-CMV-LAP_2x_NLS and pGF1-LRE-TFEB(S211A)-GFP) and co-transduced sAD2.1 iPSNs (see methods). Similar to results in N2a cells, light-exposed iPSNs displayed a significant increase in TFEB-GFP and a consequential decrease in both AT8 and AT180 p-Tau levels compared to Dark controls (Fig 5A and 5B). Lastly, it has been established that the LAP spontaneously gets inactivated in the Dark, reducing LRE-mediated gene expression [47]. Therefore, to assess the temporal dynamics of Opto-TFEB, we analyzed the light-Dark activity across two days. On day one, iPSNs was stimulated with light overnight and an identical plate of iPSNs was left in the dark. After the first time point after 12 h of light stimulation, a row of cells was collected for analysis. The following day, the light was left off and another row of cells were collected for analysis 24 h after the first collection. First, we measured the mRNA levels of three known TFEB targets; *PTEN* [29], *CTSF* [31], and *MCOLN1* [31] (Fig 5C). On day one, we observed a significant increase in TFEB expression with light and up-regulation of TFEB target genes compared to Dark (Fig 5C). The mRNA levels of TFEB-target genes reduced back to basal levels after a day of no light. Western blot analysis to detect total protein levels revealed p-Tau (AT8 and AT180) was significantly reduced (Fig 5F). Notably, while the total tau levels were unaltered, Tau12^+^ bands showed slightly faster migration (Fig 5D). On day two, levels of TFEB (S211A)-GFP and TFEB targets were down to Dark levels, however the AT8^+^ and AT180^+^ p-Tau levels seem to have gradually raised but still remained significantly lower than their starting levels (Fig 5E). Taken together, for the first time, these results suggest that light-induced, optogenetic-based expression of TFEB can reduce p-Tau in a human relevant iPSN tauopathy model.

## Discussion

Here we demonstrate the utility of an optical system to transiently regulate expression of TFEB, which is a master transcriptional regulator of autophagy to reduce the load of pathological forms of tau on neurons. We had to optimize the promoter and NLS of the original described system [43] in order to promote efficient gene expression not only in HEK293 cells, but also in N2a and iPSN neuronal models of tauopathy. We also observe that constitutively active TFEB has the capability of inducing the autophagy-mediated clearance of multiple forms of p-Tau. In addition to promoting autophagy and lysosome biogenesis, TFEB has been shown to promote a variety of biological functions including the inflammatory process [64], stress-responsive pathways [64], oxidative stress [65], and metabolic regulation [66]. Therefore, considering TFEB as a potential therapeutic target has to be a cautious move, as it cannot remain in nuclear and be constitutively active. Our study described here is aimed towards achieving the transient ‘on/off’ activation/deactivation mechanism using a novel blue light inducible TFEB gene expression system that works well in mouse neuronal cell lines and human AD iPSCs derived into mature neurons. Using diseased iPSCs can be hugely beneficially in studying because the disease phenotype is displayed when derived into another cell type [67–69]. The benefits of using sAD2.1 iPSCs is that they are derived from a patient with sporadic AD and when these iPSCs were differentiated into neurons, they display major hallmarks of AD, including elevated levels p-Tau phosphorylated at Thr231 [48]. Therefore, using sAD2.1 avoided tri-plasmid transfections, which often tend to show poor efficiency. Previous studies have utilized iPSNs to assess the role of autophagy in regulating AD-endophenotypes. For example, Reddy et al. generated human forebrain cortical neurons from iPSCs derived from familial AD patients carrying presenilin-1 (PS-1) mutations (M146L and A246E) and PS-1 knockdowns in neurons [70]. Using the same CLEAR-luciferase reporter assay as our group did, they found a reduction in CLEAR activity in the forebrain cortical iPSNs, which suggests reduction of autophagy flux. In another study, exposure of iPSC-derived forebrain cortical neurons with the amino acid metabolite homocysteine (Hcy) caused reduced autophagic activity via elevation of mTORC1 activity. Therefore, reduction in TFEB activity was suggested to be due to hyper- phosphorylation of TFEB by mTORC1 [71].

Not only have we shown successful light controlled expression of TFEB, but we also effectively enhanced the autophagy flux via mutation of mTORC1 site—S211A, which facilitated nuclear entry of TFEB and robust clearance of p-Tau in the human AD derived iPSNs. AT8 and AT180 show an increase on Day 2 (when the Light is off), which complements with notable tau buildup due to Dark (no induction of autophagy beyond basal level). Considering the reproduction of p-Tau on the day after light was turned off, proves a spatio-temporal dynamic with our Opto-TFEB system and we hypothesize when turning off autophagy, the potential kinases are likely activated again and/or likelihood of re-accumulation of hyperphosphorylated tau. However, to achieve sustained suppression of p-Tau, precise titration of light-dosage is necessary. It is also essential to induce Opto-TFEB in various time-points during the course of p-Tau pathogenesis, to assess cell toxicity besides characterizing the optimum illumination dosage of blue light to achieve precise Opto-TFEB induction to be beneficial. One of the potential limitations of inducing transcription factors is likelihood of strict regulation and compensation [72]. Furthermore, while autophagy is generally thought to promote survival as discussed above, certain conditions can lead to autophagic-mediated cell death. For instance, constitutive activation of the δ2 glutamate receptor is thought to cause Purkinje cell death in Lurcher mice via activation of autophagy processing [39]. Multiple reports demonstrate prolonged activation of autophagy proteins (e.g. LC3 and BECN1) and vacuoles in response to ischemic stroke/reperfusion *in vivo*, or oxygen-glucose deprivation (OGD) *in vitro* [73]. Interestingly, many autophagic processes do not significantly affect cell health until days after the injury, indicating that prolonged activation is critical for cell death to occur [73]. Furthermore, administration of the autophagy-inhibiting chemical 3-MA significantly reduced cell death in cells that underwent OGD [73] or ischemic injury [74]. Lastly, administration of Wortmanin reduced autophagic processing and improved memory in animals with vascular dementia [75]. Thus, for elderly tauopathy patients who may be at enhanced risk for other types of brain damage such as ischemia and vascular dementia, chronic induction of autophagy could exacerbate cell death rather than reduce it.

Nonetheless, our study demonstrates the expression and functional efficacy of neuronal Opto-TFEB in inducing the expression of CLEAR network genes for the induction of autophagy-lysosomal pathways and p-Tau clearance. It may be interesting to see if tunable Opto-TFEB expression system would work in other cell types within the CNS. Conversely, it is also important to determine whether or not such regulation is applicable to other genes of interest (example, protein phosphatases, which could dephosphorylate hyperphosphorylated tau). Moreover, our current proof-of-concept studies on Opto-TFEB specifically targeted against the shortest isoform of tau with phosphorylation-mimicking mutations (0N3R-T231D/S235D). The 0N3R tau is directly relevant to AD (as it is one of the all six isoforms of non-mutant tau, expressed in adult human brain [11,55] and numerous studies have suggested relevance of T231 site phosphorylation, including its relevance to *cistauosis* relevant to AD[14]). However, testing the efficacy of Opto-TFEB in other isoforms of tau (1N3R, 1N4R etc.) is necessary and very likely to be attempted in future studies. In conclusion, our data strongly suggest that light controlled Opto-TFEB can efficiently be expressed in AD iPSNs, subsequently up-regulates TFEB target genes, and efficiently facilitates the clearance of p-Tau. Some of the main limitations of this study are that the approach is still early in the development. For example, we wonder if light-induced regulation of autophagy can reduce p-MAPT with minimal side effects in an animal model. However, we believe our current methods/efficiency of transgene transduction is still not optimal and feasibility for *in vivo* studies. Therefore *in vivo* applications of Opto-TFEB are still questionable. Nonetheless, re-validation of this approach by other independent groups with improved efficacy may likely create a novel platform for optogenetic-based strategies to target multiple cellular signaling cascades that drive a variety of neurodegenerative (and other) diseases.

## Supporting information

S1 Fig Uncut blots for the montage shown in Fig 2A. Tau12, GFP and GAPDH specific bands (red arrows) in the uncut blots showing in Fig 2A.
(PDF)

S2 Fig Uncut blots for the montage shown in Fig 2B. Tau12, GFP and GAPDH specific bands (red arrows) in the uncut blots showing in Fig 2B. Red ‘X’ are the lanes not used in the montage shown in Fig 2B.
(PDF)

S3 Fig Uncut blots for the montage shown in Fig 4A. VP16, Tau12, GFP and GAPDH specific bands (red arrows) in the uncut blots showing in Fig 4A.
(PDF)

S4 Fig Uncut blots for the montage shown in Fig 5D. GFP, Tau12, VP16, AT8, AT180 and GAPDH specific bands (red arrows) in the uncut blots showing in Fig 5D. Red ‘X’ are the lanes not used in the montage shown in Fig 5D.
(PDF)
