# Supplementary figures and images for: Optical induction of autophagy via Transcription factor EB (TFEB) reduces pathological tau in neurons
Source: PLoS One. 2020 Mar 24;15(3):e0230026. doi: 10.1371/journal.pone.0230026 (PMC7092971; doi:10.1371/journal.pone.0230026)

FIGURE 2A

|             |   |   |   |   |   |   |   |   |   |
|-------------|---|---|---|---|---|---|---|---|---|
| WT – 0N3R   | - | + | + | - | - | - | - | - | - |
| T231D/S235D | - | - | - | + | + | - | - | - | - |
| P301L       | - | - | - | - | - | + | + | - | - |
| WT – 0N4R   | - | - | - | - | - | - | - | + | + |
| TFEB-GFP    | - | - | + | - | + | - | + | - | + |

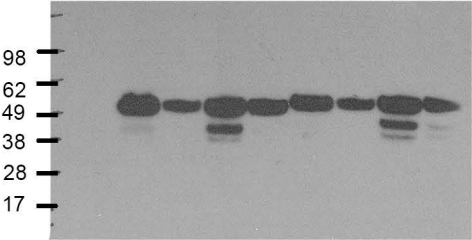

←  
**Tau12**

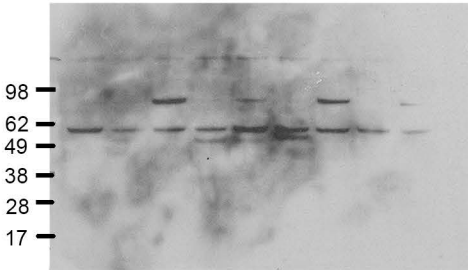

←  
**GFP**

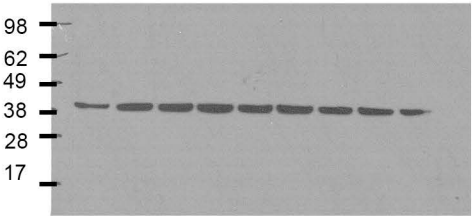

←  
**GAPDH**

|             |   |   |   |   |   |   |   |   |   |
|-------------|---|---|---|---|---|---|---|---|---|
| WT – 0N3R   | - | + | + | - | - | - | - | - | - |
| T231D/S235D | - | - | - | + | + | - | - | - | - |
| P301L       | - | - | - | - | - | + | + | - | - |
| WT – 0N4R   | - | - | - | - | - | - | - | + | + |
| TFEB-GFP    | - | - | + | - | + | - | + | - | + |

Supplement: S1 Fig — Tau12, GFP and GAPDH specific bands (red arrows) in the uncut blots showing in Fig 2A. (PDF) [file pone.0230026.s001.pdf]

**FIGURE 5D** Imaged through ChemiDoc

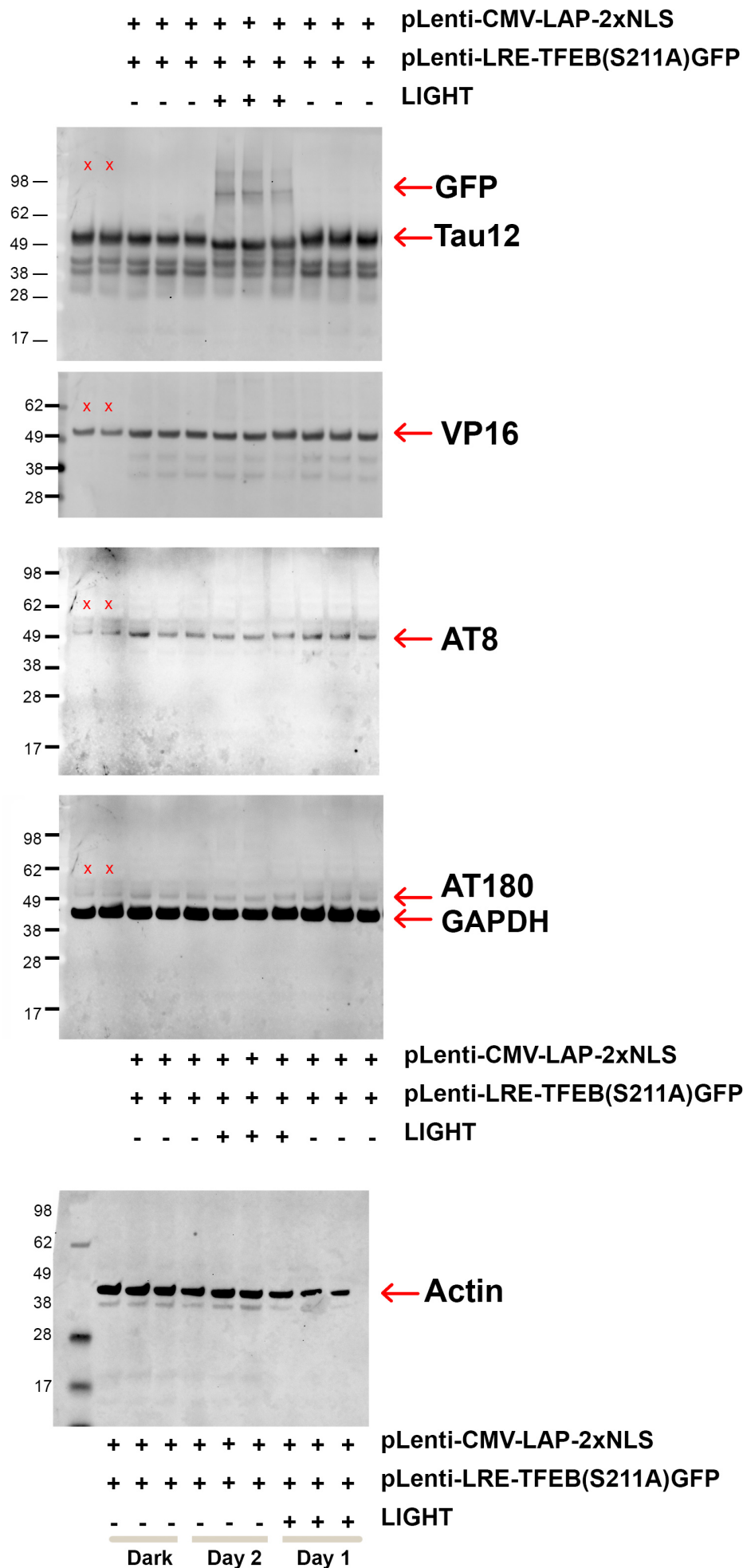

Supplement: S4 Fig — GFP, Tau12, VP16, AT8, AT180 and GAPDH specific bands (red arrows) in the uncut blots showing in Fig 5D. Red ‘X’ are the lanes not used in the montage shown in Fig 5D. (PDF) [file pone.0230026.s004.pdf]
