# Supplementary material for: Optical induction of autophagy via Transcription factor EB (TFEB) reduces pathological tau in neurons
Source: PLoS One. 2020 Mar 24;15(3):e0230026. doi: 10.1371/journal.pone.0230026 (PMC7092971; doi:10.1371/journal.pone.0230026)
Supplement: S2 Fig — Tau12, GFP and GAPDH specific bands (red arrows) in the uncut blots showing in Fig 2B. Red ‘X’ are the lanes not used in the montage shown in Fig 2B. (PDF) [file pone.0230026.s002.pdf]

FIGURE 2B

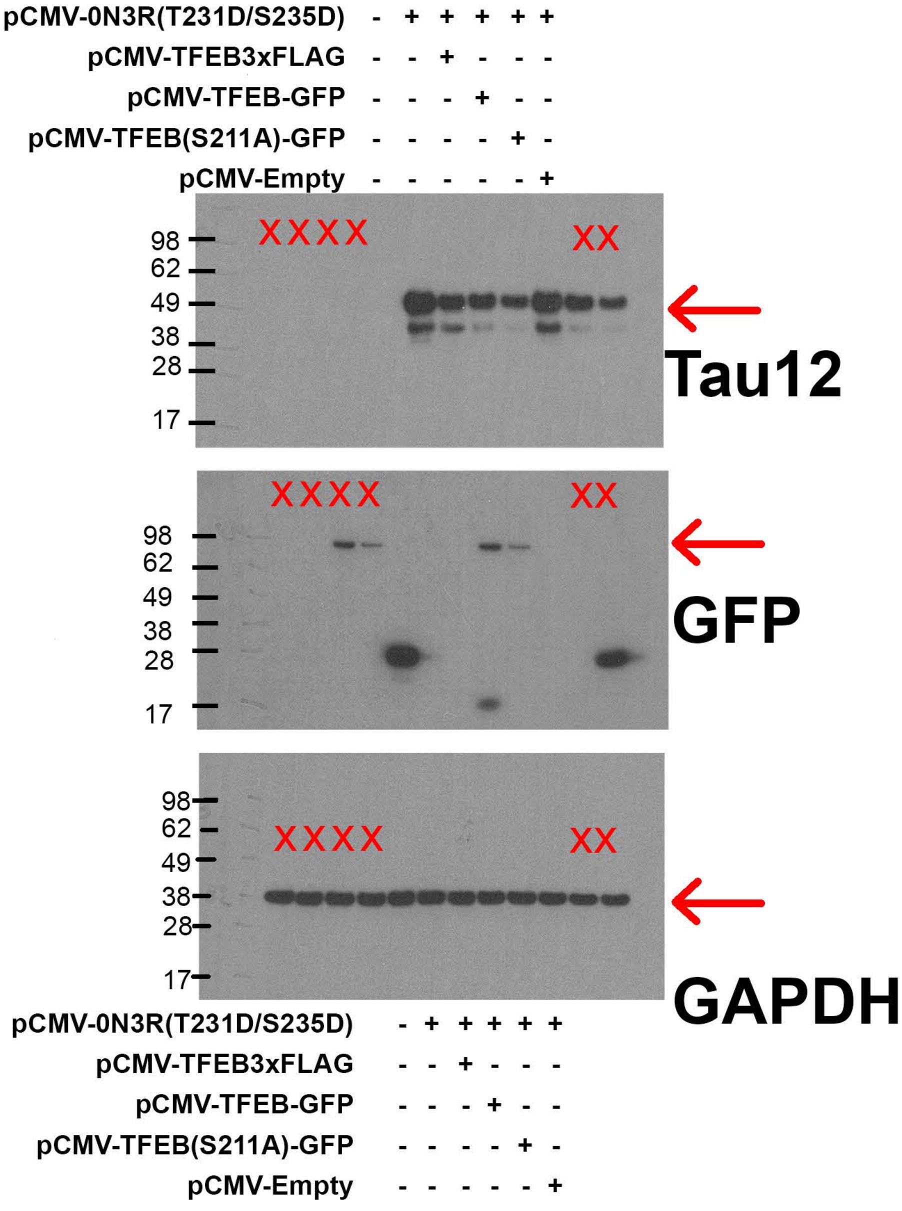
pCMV-0N3R(T231D/S235D) - + + + + +
pCMV-TFEB3xFLAG - - + - - -
pCMV-TFEB-GFP - - - + - -
pCMV-TFEB(S211A)-GFP - - - - + -
pCMV-Empty - - - - - +
XXXX
XX
98
62
49
38
28
17
Tau12
GFP
GAPDH
