# Supplementary material for: Optical induction of autophagy via Transcription factor EB (TFEB) reduces pathological tau in neurons
Source: PLoS One. 2020 Mar 24;15(3):e0230026. doi: 10.1371/journal.pone.0230026 (PMC7092971; doi:10.1371/journal.pone.0230026)
Supplement: S3 Fig — VP16, Tau12, GFP and GAPDH specific bands (red arrows) in the uncut blots showing in Fig 4A. (PDF) [file pone.0230026.s003.pdf]

FIGURE 4A Imagined through ChemiDoc

T231D/S235D	+	+	+	+	+	+	+	+
pCMV-TFEB	-	+	-	-	-	-	-	-
pCMV-LAP	-	-	+	+	+	-	-	-
LRE-TFEB lone	-	-	+	+	+	+	+	+
LIGHT	+	+	+	+	+	-	-	-

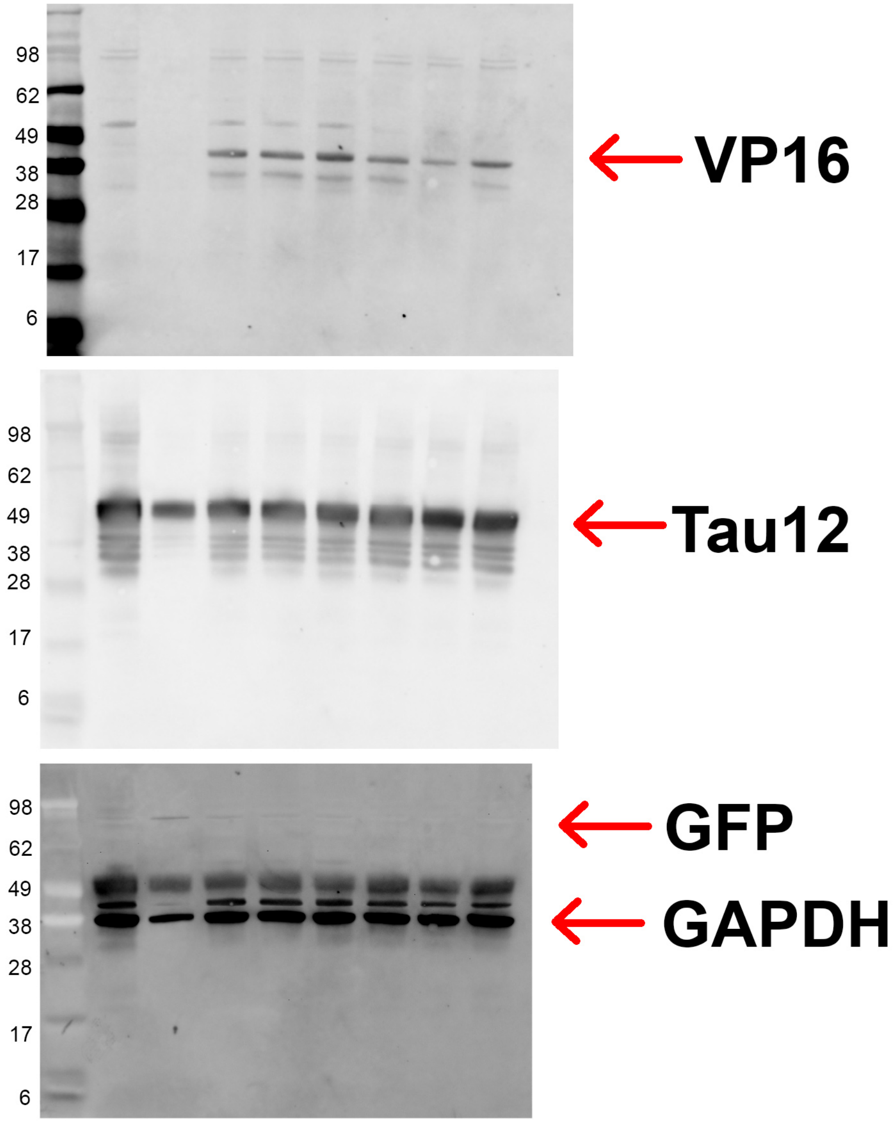

T231D/S235D	+	+	+	+	+	+	+	+
pCMV-TFEB	-	+	-	-	-	-	-	-
pCMV-LAP	-	-	+	+	+	-	-	-
LRE-TFEB lone	-	-	+	+	+	+	+	+
LIGHT	+	+	+	+	+	-	-	-
